# LP-MAB: Improving the Energy Efficiency of LoRaWAN Using a Reinforcement-Learning-Based Adaptive Configuration Algorithm

**DOI:** 10.3390/s23042363

**Published:** 2023-02-20

**Authors:** Benyamin Teymuri, Reza Serati, Nikolaos Athanasios Anagnostopoulos, Mehdi Rasti

**Affiliations:** 1 Department of Computer Engineering, Amirkabir University of Technology, Tehran P.O. Box 15875-4413, Iran; 2 Faculty of Computer Science and Mathematics, University of Passau, 94032 Passau, Germany; 3 Centre for Wireless Communications, University of Oulu, 90570 Oulu, Finland

**Keywords:** Internet of Things (IoT), LoRaWAN, adaptive configuration, machine learning, reinforcement learning

## Abstract

In the Internet of Things (IoT), Low-Power Wide-Area Networks (LPWANs) are designed to provide low energy consumption while maintaining a long communications’ range for End Devices (EDs). LoRa is a communication protocol that can cover a wide range with low energy consumption. To evaluate the efficiency of the LoRa Wide-Area Network (LoRaWAN), three criteria can be considered, namely, the Packet Delivery Rate (PDR), Energy Consumption (EC), and coverage area. A set of transmission parameters have to be configured to establish a communication link. These parameters can affect the data rate, noise resistance, receiver sensitivity, and EC. The Adaptive Data Rate (ADR) algorithm is a mechanism to configure the transmission parameters of EDs aiming to improve the PDR. Therefore, we introduce a new algorithm using the Multi-Armed Bandit (MAB) technique, to configure the EDs’ transmission parameters in a centralized manner on the Network Server (NS) side, while improving the EC, too. The performance of the proposed algorithm, the Low-Power Multi-Armed Bandit (LP-MAB), is evaluated through simulation results and is compared with other approaches in different scenarios. The simulation results indicate that the LP-MAB’s EC outperforms other algorithms while maintaining a relatively high PDR in various circumstances.

## 1. Introduction

The Internet of Things (IoT) refers to the network of physical objects—“things”—embedded with sensors and software that use the internet to transmit and receive data. With several billion connected IoT devices today, experts expect this number to grow to 22 billion by 2025 [1]. Various requirements are essential to IoT applications, including a long transmission range, low energy consumption, and a cost-effective design. Short-range communication technologies, such as Bluetooth, ZigBee, and Wi-Fi, are unsuitable for long-range communication. In contrast, cellular communication networks, such as 3G and 4G cellular networks, can provide a much wider transmission range at the expense of draining the ED’s battery quickly. Therefore, in order to meet the needs of IoT applications, long-range and low-power networks are required. Low-Power Wide-Area Networks (LPWANs) are wireless networks that enable long-range communications with low data rates and low energy consumption. An LPWAN can provide a transmission range of 40 and 10 kilometers (km) in sub-urban and urban environments, respectively [2], with a maximum battery life of ten years [3].

**Figure 1:**
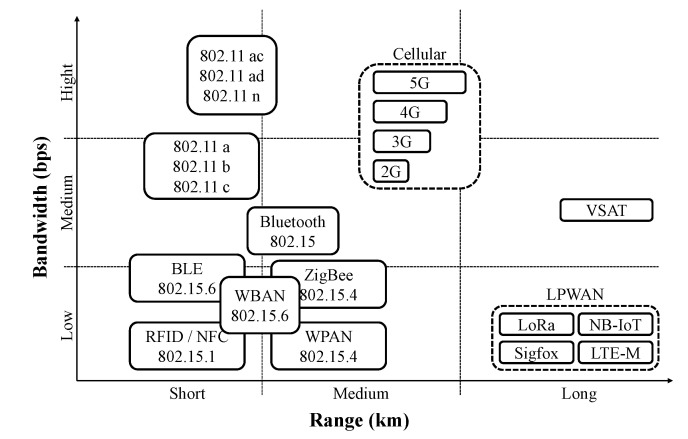
Range of wireless protocols, according to [10] and our own knowledge and experience.

As shown in Figure 1, compared with other technologies, LPWAN was mainly designed for IoT applications that require the non-periodic transmission of short messages in extended radio coverage. LPWANs can operate on both licensed and unlicensed frequencies, such as LoRa [4], NarrowBand IoT (NB-IoT) [5,6], Sigfox [7], and LTE-M [8]. LoRa (short for **Lo**ng **Ra**nge) is a physical proprietary radio communication technique that utilizes spread-spectrum modulation derived from the Chirp Spread Spectrum (CSS) technology. LoRa enables long-range and low-power communication, where packets of up to 256 bytes can be sent with each message transfer at sub-GHz frequencies [9]. A number of network evaluation criteria can be used to assess the efficiency of LPWANs, including Packet Delivery Ratio (PDR) and Energy Consumption (EC).

In the LoRa network, several transmission parameters must be configured before a connection can be established. These transmission parameters are: Spreading Factor (SF), Transmission Power (TP), Carrier Frequency (CF), Coding Rate (CR), and BandWidth (BW). Various values can be assigned to each of these parameters. By configuring each transmission parameter to a different value, a relatively large state space of configurations, consisting of several hundreds of states, exists. The selection of each of these states can affect the network evaluation criteria, such as PDR and EC. As an example, if ED transmits with SF7 and TP2, i.e., using the minimum spreading factor and transmission power, respectively, the least noise sensitivity, the lowest transmission delay, and the least coverage area are achieved. In this regard, it is crucial to find the optimal configuration of the transmission parameters [11].

The Adaptive Data Rate (ADR) algorithm is a mechanism to adjust the transmission parameters of LoRa EDs with the objective of improving the values achieved for the network evaluation criteria. This mechanism was first proposed in the LoRaWAN specification v1.1 [4]. In this mechanism, the Network Server (NS) uses the highest Signal-to-Interference-plus-Noise Ratio (SINR) for link quality assessment after receiving the last 20 packets from each ED.

Machine Learning (ML) algorithms, which provide a self-learning process, are divided into supervised, unsupervised, and Reinforcement Learning (RL). There is no need for training data sets in RL-based methods, as learning happens through interaction with the environment. The RL agent can perceive and analyze its environment, take actions, and learn through trial and error [12]. Therefore, RL-based methods are the best choice for low-complexity network deployment. The literature review shows that RL techniques can improve resource allocation performance in LoRaWAN by allowing each ED to select the most appropriate configuration of transmission parameters.

In [13,14], an RL-based non-stationary resource allocation algorithm called LoRa-MAB is proposed, based on an adversarial environment suitable for LoRaWAN deployments. However, as a result of the long exploration process of the approach, LoRa-MAB experiences a high EC. Moreover, in the distributed solutions, the transmission parameters configuration is done on the EDs’ side, which are resource-constrained devices that are not designed to handle the computational overhead. Since many IoT applications employ battery-powered EDs that are used in large numbers for lengthy periods of time, EC plays an essential role in measuring the performance of such algorithms.

To meet the desired communication performance, it is challenging to determine the proper configuration of the transmission parameters so that EC is minimized and PDR is enhanced. In our previous works, [15,16], we have used centralized and distributed ADR approaches, respectively, to find a solution to this problem. In [15], a low-complexity ADR scheme was proposed, in which the NS attempts to obtain the optimal transmission parameters of the EDs, not by considering the history of the last 20 packets received, but by considering only the current environmental conditions of the communication, based on the transmission parameters of the last packet received. In [16], each ED individually tries to find the optimum transmission parameter configuration with the help of ML approaches.

This article focuses on improving the EC by combining non-stationary adversarial algorithms, suitable for the LoRa environment, with stochastic algorithms, which have the advantage of a short exploration time. Our work also reduces the overall computational overhead by migrating the implementation of the scheme to the NS without imposing any changes on the protocol design. Thereby, we improve upon our previous work that has been presented in [16], by reducing the overall EC and improving the PDR. The main contributions of our article can be summarised as follows:

- In this paper, we propose a **L**ow-**P**ower **M**ulti-**A**rmed **B**andit (henceforth, LP-MAB) ADR mechanism, a centralized adaptive configuration scheme in LoRaWAN. In particular, we employ EXPonential weights for EXPloration and EXPloitation (EXP3) along with the Successive Elimination (SE) technique. As a result, the proposed solution combines non-stationary adversarial and stochastic methods.
- In order to assess the performance of LP-MAB, simulation results for LP-MAB and various other ADR schemes, namely, ADR-MAX [4], ADR-AVG [11], No-ADR, and ADR-Lite [15], have been compared. These results indicate that the LP-MAB’s EC outperforms other algorithms while maintaining a relatively high PDR in various circumstances, considering both stationary and mobile EDs. This is achieved by determining the effects of various parameters and conditions such as channel noise, simulation time, network size, and the number of daily sent packets by each ED. We also consider both an urban and a sub-urban environment for all the examined scenarios, while also studying the impact of network densification, i.e., the number of EDs in the simulation.

In general, this article follows the following structure: Background information and related works are presented in Section 2 and Section 3, respectively. Section 4 describes our LP-MAB algorithm. Then, the simulation setup and our results are presented in Section 5 and Section 6, respectively. Finally, Section 7 concludes this work.

## 2. Background

In this section, following a review of the LoRa and LoRaWAN protocol stack, an EC model will be discussed for LoRa EDs, since we are primarily concerned with optimizing the energy consumption of the LoRa network. This section will be concluded with a thorough description of the Adaptive Data Rate (ADR) mechanism.

### 2.1. LoRa Overview

**Figure 2:**
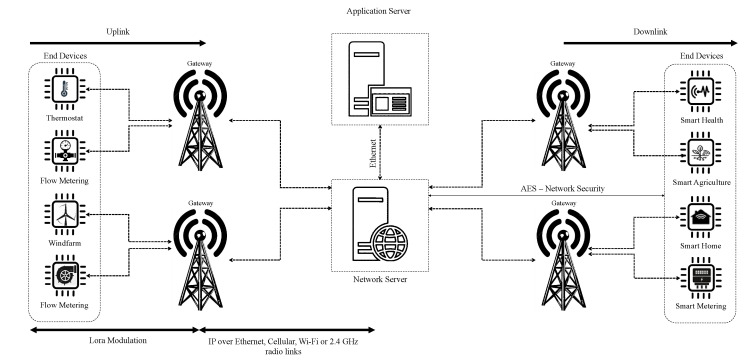
LoRaWAN network architecture.

The LoRa architecture is based on a star-of-stars topology, consisting of four components, i.e., EDs, GateWays (GWs), the NS, and an application server, as shown in Figure 2. Multiple GWs are located at different locations to receive the uplink data from EDs. As uplink messages are broadcast over the network, EDs are not assigned to a specific GW. The received LoRa packets by the GW, are then relayed to the NS over a backbone network, which can, for example, be implemented using IP over Ethernet, cellular, Wi-Fi, or 2.4-GHz radio communication. Packets are then routed to the relevant application by the NS, e.g., by using an Ethernet connection. Both uplink communication (ED to application) and downlink communication (application to ED) can be performed by the NS. The LoRa specification is documented in more detail in [4].

In LoRa, communication link quality is impacted by several transmission parameters [17], which are:

- ***SF:*** SF can be described as the number of symbols that can appear in a single bit of transferred data, which can be set in the range of 7 to 12, depending on the environmental conditions between the ED and the GW.
- ***TP:*** The TP of LoRa radio can be configured between 2 dBm and 14 dBm in steps of 3 dBm (the setup of each transmitter may vary). When the TP increases, the signal range increases while the battery lifetime of the EDs shortens, and vice versa.
- ***CF:*** Lower frequency ranges result in decreased receiver antenna size, BW capacity, and latency, while increasing the coverage. A LoRa communication network can operate over radio frequency bands below 1 GHz, including frequencies of 433, 868, and 915 MHz, with different step sizes depending on the regulation rules.
- ***CR:*** CR improves LoRa communication link robustness with Cyclic Redundancy Check (CRC). The values of CR may vary in the range of $\left\{\frac{4}{5},\frac{4}{6},\frac{4}{7},\frac{4}{8}\right\}$.Adding such error correction coding will increase the transmission overhead, which can affect the performance.

Various parameters and conditions, such as BW, channel noise, simulation time, network size, packet length, ED speed, and the number of daily sent packets by each ED, can affect the network performance. The value of BW, in particular, may vary in the range of {125,250,500} kHz.

### 2.2. An EC Model for LoRa EDs

**Figure 3:**
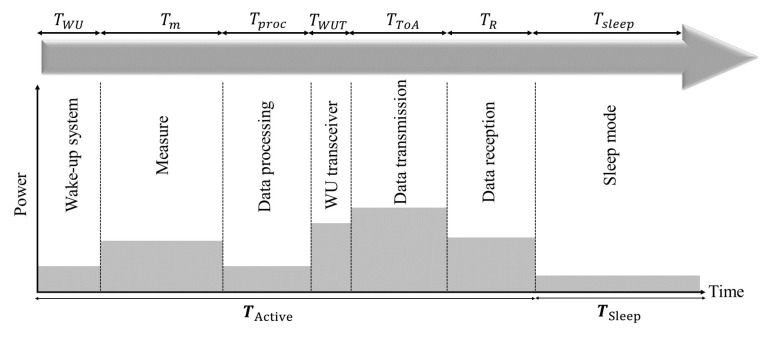
The assumed working mode sequence for each ED, adopted from [18].

To have a realistic EC model for LoRa EDs, we assume the same sequence of working modes for the LoRa and LoRaWAN sensor nodes as the one presented in [18]. This sequence of working modes is illustrated in Figure 3. Thus, the total energy consumed by the EDs, $E_{\mathrm{Total}}$, is calculated as follows:

(1) $$E_{\mathrm{Total}}=E_{\mathrm{Sleep}}+E_{\mathrm{Active}}\;,$$

where $E_{\mathrm{Sleep}}$ and $E_{\mathrm{Active}}$ is the energy consumed by the EDs during the sleep and the active modes, respectively. The total EC of EDs in the active mode is calculated by the summation of the energy consumed during the relevant working modes of the EDs (from the ones shown in Figure 3). Thus, $E_{\mathrm{Active}}$ is calculated as shown in the following equation [19]:

(2) $$E_{\mathrm{Active}}=E_{WU}+E_{m}+E_{\mathrm{proc}}+E_{WUT}+E_{ToA}+E_{R}\;.$$

Hence, in Equation (2), $E_{WU}$, $E_{m}$, $E_{\mathrm{proc}}$, $E_{WUT}$, $E_{ToA}$ and $E_{R}$, describe the EC of the wake-up of the device, the data measurement, the microcontroller processing, the LoRa transceiver’s wake-up, the transmission, and the reception mode, respectively, as shown in Figure 3. The consumed energy in the data transmission mode, $E_{ToA}$, is expressed as follows [18]:

(3) $$E_{ToA}=\left(P_{ON}\left(f_{MCU}\right)+P_{ToA}\right)\times T_{ToA}\;.$$

Here, $P_{ON}\left(f_{MCU}\right)$ is the microcontroller’s EC depending on its processor frequency $f_{MCU}$, while $P_{ToA}$ and $T_{ToA}$ are the consumed power in the transmission mode and its time duration, respectively [18]. The power utilization of LoRa sensors in the active mode depends on the Time-on-Air (ToA) duration. An ED requires time to transfer both the preamble and the payload message, i.e., $T_{\mathrm{Preamble}}$ and $T_{\mathrm{Payload}}$, respectively [19], which leads to the following equation:

(4) $$T_{\mathrm{ToA}}=T_{\mathrm{Preamble}}+T_{\mathrm{Payload}}\;.$$

$T_{\mathrm{Preamble}}$ can be obtained as follows:

(5) $$T_{\mathrm{Preamble}}=\left(4.25+N_{P}\right)\times T_{\mathrm{Symbol}}\;.$$

Let the number of preamble symbols be $N_{P}$, and the symbol’s length be denoted by $T_{\mathrm{Symbol}}$, which is defined as the duration time for transmitting $2^{SF}$ chirps. Note that the BW is equal to the chirp rate. The symbol duration is calculated so that:

(6) $$T_{\mathrm{Symbol}}=\frac{2^{SF}}{BW}\;.$$

Moreover, $T_{\mathrm{Payload}}$ (in seconds) is calculated using this equation:

(7) $$T_{\mathrm{Payload}}=T_{\mathrm{Symbol}}\times N_{\mathrm{Payload}}\;.$$

$N_{\mathrm{Payload}}$ is the number of symbols transmitted as message payload, except the preamble, specified as [19]:

(8) $$N_{\mathrm{Payload}}=8+\max\left(\left\lceil \frac{\Theta (PL,SF)}{\Gamma (SF)}\right\rceil \times \frac{1}{CR},0\right)\;.$$

We use the following equation to calculate Θ(PL,SF):

(9) Θ(PL,SF)=8×PL−4×SF+16+28−20×H.

In this equation, *H* is zero when the header is enabled, and *H* is equal to one when there is no header present. Γ(SF) can be calculated as SF−2×DE, wherein DE is set as one when the low data rate optimization is enabled; otherwise, DE is set to zero.

As observed from Equations (1)–(9), higher SF values significantly increase the EC: Higher SF values exponentially increase $T_{\mathrm{Symbol}}$ (Equation (6)), leading to long $T_{\mathrm{Preamble}}$ (Equation (5)) and, thus, $T_{ToA}$ (Equation (4)). The increase in $T_{ToA}$ then leads to $E_{ToA}$ being higher (Equation (3)), making the $E_{\mathrm{Active}}$ (Equation (2)) and $E_{\mathrm{Total}}$ (Equation (1)) larger. Therefore, compared to lower SFs, transmitting the same amount of data with a higher SF requires a much higher $T_{ToA}$ and, thus, a much higher EC.

### 2.3. The ADR Mechanism

There are two methods to control the transmission parameters in LoRaWAN: distributed and centralized approaches [11]. In the distributed method, each ED tries to configure its own transmission parameters based on the NS’s ACKnowledgment (ACK) regarding the reception or non-reception of the uplink messages, e.g., in the ADR-AVG [11], and ADR-Lite [15] schemes. In the centralized method with a global knowledge of the network, the NS tries to configure the transmission parameters of each link according to the ACK messages individually, e.g., in the MIX-MAB [16], and LoRa-MAB [13] schemes. Our work significantly extends and revises the MIX-MAB work.

The Adaptive Data Rate (ADR) algorithm is a mechanism to configure the transmission parameters of EDs with the aim of improving PDR and EC as the two primary performance metrics. Through a centralized manner of configuring the EDs’ transmission parameters, ADR aims to optimize data rate and ED lifetime. ADR, for this purpose, evaluates the link-budget estimation between EDs and GWs in the uplink messages. In this regard, Media Access Control (MAC) commands will be used to control the data rate of the ED if the ADR bit is set. Each ED and the NS may set and unset the relevant ADR bit on demand. When the ADR bit is not set, the NS will not configure the ED’s transmission parameters, regardless of the signal quality received by the end device. However, the ADR scheme should be enabled whenever possible to maximize the network capacity and battery lifetime of EDs. More details about the ADR mechanism are provided in [20,21].

## 3. Related Works

There have been several studies to improve LoRaWAN performance, focusing on statistical and mathematical models [22], the effect of the number of GWs [23], optimization algorithms [24,25], and machine learning techniques [16]. Configuring the LoRaWAN transmission parameters to address scalability has been presented in [11]. In recent years, the ADR approach has been proposed in version 1.1 of the LoRaWAN Specification [4]. In [4], the maximum value of the latest twenty received packets’ Signal to Interference and Noise Ratio (SINR) is taken into account as an indicator to evaluate the link quality. However, in this optimistic approach, environmental changes cannot realistically be considered.

The proposed methods in [11] and [24] improved the original ADR mechanism by using the details, i.e., the SINR, of the last 20 received packets to adjust the transmission parameters. More specifically, by using the average SINR value of the last twenty packets in [11], in a method called ADR-AVG, instead of the maximum value of the SINR, resulted in better performance. In [24], the authors proposed a new ADR, called ADR-OWA, using the Ordered Weighted Averaging (OWA) function. However, when channel saturation in either an urban or a sub-urban environment is low, the EC of ADR-OWA will be higher than ADR-AVG.

In addition to the emerging ML techniques, the new IoT ED requirements for more reliability, as well as low latency demands, led to the development of more efficient optimization mechanisms [14]. Self-resource management is critical to improving the battery lifetime for LoRa EDs. Some works focused on using ML techniques, such as RL, to enable EDs to use innovative and inherently distributed techniques for the management of the transmission parameters [13,14]. MAB [13,14] and Q-learning [26] are two RL algorithms used in the literature to propose distributed radio resource allocation in LoRaWAN. In [26], the authors use RL by offering a Q-learning model combined with Carrier-Sense Multiple Access with Collision Avoidance (CSMA/CA), to decrease the collision rate and improve the PDR. However, in addition to the increased EC using the method in [26], Q-Learning requires a database to save its processing data, a requirement that is not compatible with resource-constrained IoT EDs.

The LoRa-MAB algorithm proposed in [13,14] is based on EXP3. As a non-stationary adversarial method, this approach suffers from a rather long exploration process resulting in high EC. In particular, in the aforementioned distributed approach, the EDs and the NS must frequently communicate, resulting in reducing the battery lifetime. SE is a non-stationary stochastic MAB-based algorithm presented in [27], for which, however, the adversarial environment of LoRa has not been taken into account.

## 4. Our Proposed LP-MAB Algorithm

This section proposes a centralized adaptive configuration algorithm in LoRaWAN. In our newly proposed RL-based adaptive configuration algorithm, the NS does not need to be provided with a predefined dataset, as it will learn by interacting with the EDs. In LP-MAB, the agent is the NS interacting with the environment, including EDs, to perform an action that can be defined as the determination of the set of transmission parameters to which an ED should be configured. The NS tries to achieve the optimum action, i.e., the optimal set of transmission parameters, for each ED by learning based on the relevant reward, which is based on the reception of the ACK messages. More specifically, a LoRa ED configures its transmission parameters based on the NS’s selected action. If the NS receives the packet, it sends back a confirmation ACK message to the ED, assigns a reward (which will be defined later) to the selected action, and uses it for the subsequent transmission parameters’ index. We model the adaptive configuration scheme utilised by the NS as a MAB problem, an RL-based technique, and formulate it using *k* multi-armed bandits, where *k* represents the total configuration’s state space. An agent selects from *k* different actions and, each time, receives a reward based on its chosen action.

Three general categories, of stochastic, adversarial, and switching bandit algorithms, can be used to address the MAB problems. EXP3 is a category of non-stationary adversarial MAB problems. LoRa can be placed in this category because the selection by two or more EDs of the same transmission parameter values, such as an equal SF, affects the transmission performance of all the relevant EDs. Stochastic MAB algorithms such as SE are unsuitable for LoRaWAN due to its adversarial nature. The long exploration process of EXP3 results in a high convergence time. On the other hand, the SE algorithm has the advantage of short-term exploration. So, inspired by the benefits of the EXP3 and SE algorithms used in [13,14] and [27], respectively, we combine these two approaches and propose a new algorithm called LP-MAB.

**Figure 4:**
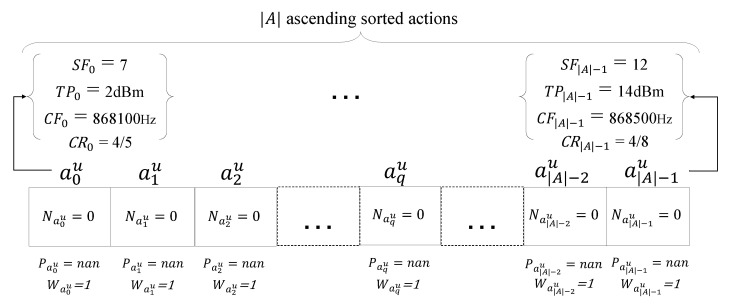
The initialization of LP-MAB for the *u*th ED.

As can be seen in Algorithm 1, at the beginning, we assume there are |U| EDs in the simulation forming the set $U=\{ED_{1},ED_{2},\ldots ,ED_{U}\}$. NS aims to maximize the PDR of the network, while keeping the EC at the minimum possible value by learning to select the optimum transmission parameter set for ∀u∈U. Assuming that each action is a vector of four transmission parameters, $a_{k}^{u}=\left\{SF_{k},TP_{k},CF_{k},CR_{k}\right\}$ denotes the *k*th action for the *u*th ED, in which $SF_{k},TP_{k},CF_{k},$ and $CR_{k}$ are the values of SF, TP, CF, and CR in the *k*th action, respectively. We assume that there are |A| actions, whose set is denoted by $A=\{a_{0}^{u},a_{1}^{u},\cdots ,a_{|A|-2}^{u},a_{|A|-1}^{u}\}$. LP-MAB allows configuring the CF and CR in addition to the SF and TP, unlike [4,11,24], making the action set size |A| rather large. Actions are sorted in A in ascending order based on their EC according to Equation (8). Let $N_{a_{k}^{u}}$ indicate how many times the NS selects the *k*th action for the *u*th ED. $W_{a_{k}^{u}}\left(t\right)$ and $P_{a_{k}^{u}}\left(t\right)$ are the weight of the *k*th action and the probability of selecting the *k*th action for the *u*th ED at the simulation time of transmission period *t*, respectively. The transmission period *t* is initialised to zero. Thus, the visual representation of Algorithm 1 can be seen in Figure 4.
**Algorithm 1:** Initialization of LP-MAB.
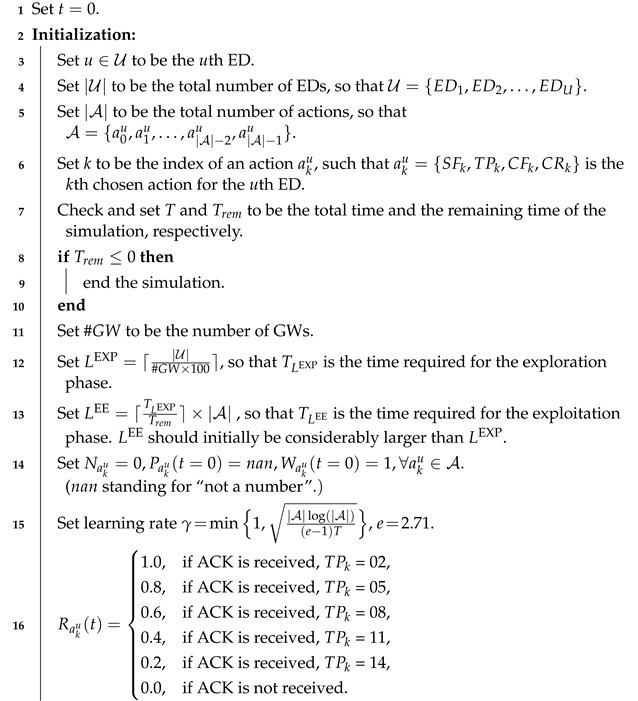


Our proposed LP-MAB algorithm is composed of two phases, exploration and exploitation, as described in Section 4.1 and Section 4.2, respectively.

### 4.1. Exploration Phase of the LP-MAB Algorithm

**Figure 5:**
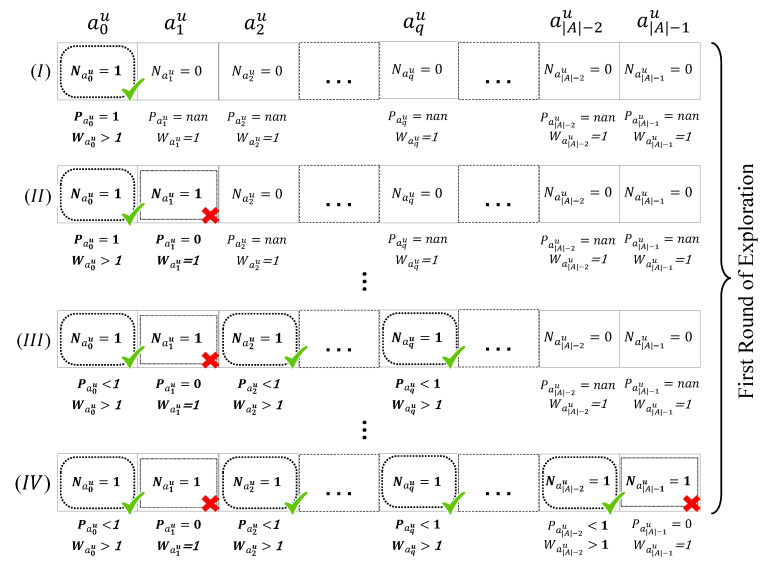
Possible first round of the LP-MAB exploration phase for the *u*th ED.

The goal of the first phase of our algorithm is to update the values of the $W_{a_{k}^{u}}\left(t\right)$ and $P_{a_{k}^{u}}\left(t\right)$ so that, in the exploitation phase, the NS can select the optimum configuration for the *u*th ED based on the information gathered from the environment. We assign probabilities to each action to obtain their weights (lines 10–12 of Algorithm 2), so that we can make a trade-off between exploration and exploitation. At the start of the simulation, the NS selects the first action for the *u*th ED, i.e., $a_{k=0}^{u}$, and then increase the value of $N_{a_{k}^{u}}$ by one, as shown in Figure 5 (I). After the ACK reception or non-reception for the chosen action (demonstrated in Figure 5 by ✓ and ✗, respectively), the NS updates the reward value, $R_{a_{k}^{u}}\left(t\right)$, based on line 16 of Algorithm 1. In this multi-reward strategy, the reception of the ACK for the action with the highest TP (TP=14), will be far less rewarded than the reception of the ACK for the action with the lowest TP (TP=2), aiming to minimize the EC as much as possible.
**Algorithm 2:** Exploration Phase of LP-MAB.
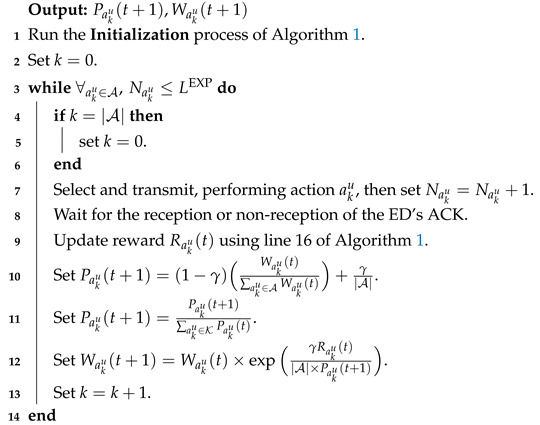


**Figure 6:**
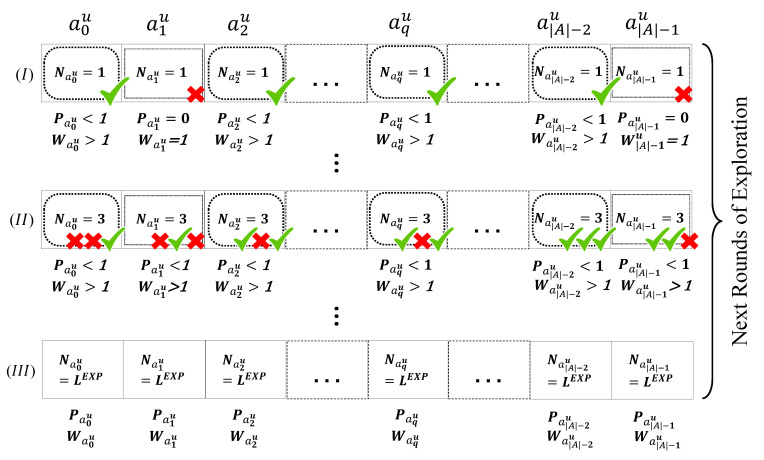
Possible next rounds of the LP-MAB exploration phase for the *u*th ED.

After calculating the reward, the NS updates the weight and probability of the action according to the lines 10–12 of Algorithm 2, which have been extracted from the EXP3 algorithm. As a rule, the summation of all probabilities is equal to one. So the action’s probability should be normalized (line 11 of Algorithm 2). For the next packet of the *u*th ED, the NS selects the action $a_{1}^{u}$ (Figure 5 (II)). This procedure for the *u*th ED continues until the NS has selected all the actions once (Figure 5 (IV) and Figure 6 (I)).

The exploration phase of the LP-MAB scheme was adopted from the SE algorithm. As can be seen in line 3 of Algorithm 2, this phase is repeated for multiple rounds, up to the $L^{\mathrm{EXP}}$ value, for each ED. A novel feature of our work in comparison to [16], is that we take into account, in the exploration phase, the potential occurrence of environmental changes, such as changes in the number of EDs and GWs, through the utilisation of $L^{\mathrm{EXP}}$. According to line 12 of Algorithm 1, $L^{\mathrm{EXP}}$ can be calculated by dividing the total number of EDs by the product of the multiplication of the number of GWs by the constant value 100. The use of this constant value is intended to reduce the value of $L^{\mathrm{EXP}}$, which should not be too large, as it would significantly prolong the exploratory phase.

In general, as the number of EDs increases, the level of interference in a fixed network area grows higher, and thus it becomes necessary to extend the exploration phase, which is achieved through the use of $L^{\mathrm{EXP}}$, the value of which is directly proportional to the number of EDs. According to [28], the reception probability in LoRa networks is dependent on the number of GWs. Thus, in case of a high number of GWs, a shorter exploration phase is needed, which is achieved by $L^{\mathrm{EXP}}$ being inversely proportional to the number of GWs. As seen in Figure 6 (III), after $L^{EXP}$ rounds, at the end of the exploration phase, the value of $N_{a_{k}^{u}}\;\left(\forall a_{k}^{u}\in A\right)$ is equal to $L^{\mathrm{EXP}}$. The weights and probabilities at the end of this phase for all the actions, will form the input of the exploitation phase.

### 4.2. Exploitation Phase of the LP-MAB Algorithm

In this phase, the actions are selected based on the relevant Probability Density Function (PDF), i.e., their probability, $P_{a_{k}^{u}}$ at the end of the exploration phase (line 2 of Algorithm 3). According to this step, which is derived from the EXP3 scheme, it is more likely that actions of high probability will be selected, i.e., more suitable actions have a higher chance to be selected. Same as in the exploration phase, also in this phase, both in the case of a successful ACK reception and in the case of a non-reception, in addition to the $N_{a_{k}^{u}}$ increment (line 3 of Algorithm 3), the NS will update the weight and probability of the relevant action (lines 6–8 of Algorithm 3).
**Algorithm 3:** Exploitation Phase of LP-MAB.
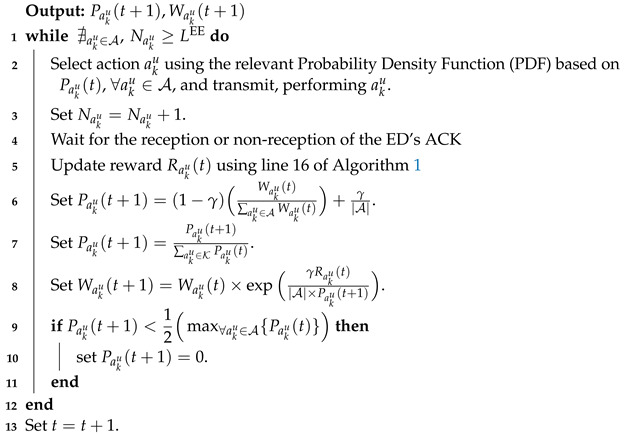


Let us consider the case in which the probability of the *k*th action for the *u*th ED, i.e., of $a_{k}^{u}$, is smaller than the half of the maximum probability when all actions in A are considered. In that case, the NS will set the probability of action $a_{k}^{u}$ to zero, so that action $a_{k}^{u}$ will not be selected until the end of the exploitation phase (lines 9–11 of Algorithm 3). This removal process aims to eliminate actions with a low probability of leading to a successful transmission. It should be noted that the above threshold (line 9 of Algorithm 3) is derived heuristically from our simulation results, leading to the best performance.

The exploitation phase for the *u*th ED continues until the number of selections of at least one of the actions ($N_{a_{q}^{u}}$, for that action $a_{q}^{u}$) reaches the value of $L^{\mathrm{EE}}$ (line 1 of Algorithm 3). Thus, $L^{\mathrm{EE}}$ should be considerably larger than $L^{\mathrm{EXP}}$ for the first few transmission periods *t* considered, as $\forall_{a_{k}^{u}\in A},\;N_{a_{k}^{u}}=L^{\mathrm{EXP}}$ at the beginning of the exploitation phase.

Our work differs from [16], in that we incorporate what we have learned from the environment during the exploration phase, through the use of the dynamic value of $L^{\mathrm{EE}}$. According to line 13 of Algorithm 1, $L^{\mathrm{EE}}$ is calculated by multiplying the total number of actions by the quotient of the division of the exploration phase duration by the remaining time of the simulation. In general, the higher the number of actions is, the higher the number of successful transmissions, i.e., of potentially optimal configurations, will be, and, therefore, the NS will need to consider more actions to select the one that reduces interference the most; thus, a longer period of time should be spent on exploitation in this case. Moreover, due to the fact that our learning about the network increases as we get closer to the end of the simulation, to utilize the information obtained during the exploration phase more effectively, it is reasonable that the exploitation phase should take longer to finish.

**Figure 7:**
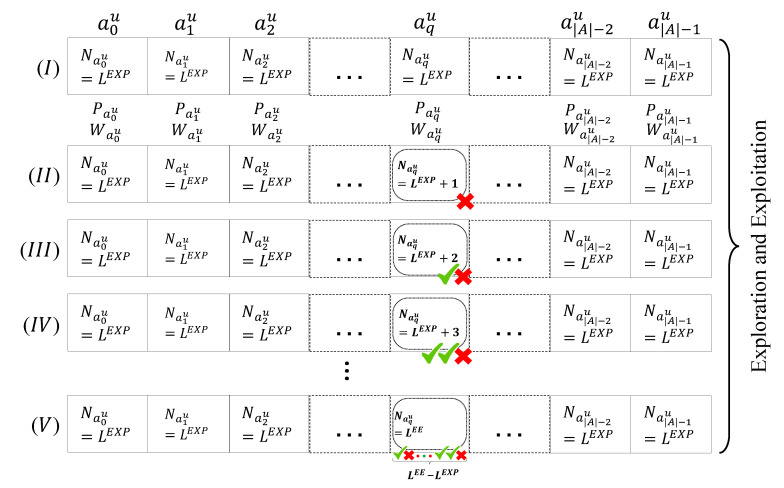
Possible LP-MAB exploitation phase for the *u*th ED, with (V) representing the final round of the exploitation phase for this transmission period of the *u*th ED. In this extreme case used as an example, action $a_{q}^{u}$ has been selected to be performed in all rounds.

As seen in Figure 7, for any arbitrary $a_{q}^{u}$, when $N_{a_{q}^{u}}$ reaches the value of $L^{\mathrm{EE}}$, the exploitation phase will be ended. At the end of this phase, the transmission period index *t* is incremented by one, so that the exploration phase can start again for a new transmission period. In this way, the actions that were removed from the previous execution will have a second chance. Note that all actions will have their $N_{a_{k}^{u}}$ set to zero, with no change in their weights or probabilities (line 14 of Algorithm 1). By not resetting the weight and probability values of the actions at the end of the exploitation phase, the previously gained knowledge is not eliminated by the proposed LP-MAB algorithm. Nevertheless, the weights and probabilities of all actions at the end of the new exploration phase, which correspond to the new transmission period, will be inputs for the exploitation phase corresponding to that transmission period.

## 5. Simulation Setup

We have used FLoRa [11] (a Framework for LoRa simulations) as a simulator tool. FLoRa, which is based on OMNeT++ [29], a discrete event network simulator, was proposed for the simulation of a LoRaWAN composed of EDs, GWs, and an NS according to the setup presented in [11]. More information regarding FLoRa is available at https://flora.aalto.fi/ (accessed on 11 January 2023). A LoRa link behavior model that considers the capture effect and inter-SF collisions in multiple network settings is presented in FLoRa. We customized FLoRa for simulating our adaptive configuration scheme based on artificial intelligence methods in the LoRa network. The LP-MAB framework is available at the following GitHub repository: https://github.com/reza-serati/LP-MAB (accessed on 11 January 2023).

We consider a LoRaWAN consisting of arbitrary numbers of GWs randomly placed in a square-shaped cell having different radius sizes based on urban and sub-urban environments, with up to 700 EDs uniformly distributed [11]. Simulations were conducted under the impact of the capture effect and inter-SF collisions to minimize the number of collisions [13]. The list of parameters that affect the performance of LoRaWAN are summarized in Table 1. It should be noted that, for the final results, a series of simulations was performed twenty times, and the resulting data were averaged.

To evaluate the proposed algorithm’s performance and compare it with other schemes, we use the following two metrics:

1. **PDR (%)**: Defined as the total number of packets received by the NS divided by the total packets sent from all EDs during the simulation time.
2. **EC (kJ)**: Defined as the total EC divided by PDR as discussed in [15].

We consider the following eight scenarios:

- ***Scenario 1:*** The number of static EDs varies from 100 to 700, with a step size of 100.
- ***Scenario 2:*** Based on the environmental conditions, for 100 static EDs, the channel saturation varies according to the values shown in Table 1.
- ***Scenario 3:*** Considering that EDs are mobile, the number of nodes varies between 100 and 700.
- ***Scenario 4:*** For 100 mobile EDs, the mobility speed can be changed using the values shown in Table 1.
- ***Scenario 5:*** Comparatively to Scenarios 3 and 4, in which all nodes were mobile, two types of EDs were considered in the simulation environment: static and mobile, for varying network sizes.
- ***Scenario 6:*** In contrast to the constant 12-day simulation time assumed in other Scenarios, in this Scenario, simulation days vary from 12 to 120, with a step size of 12 days, for 100 static EDs.
- ***Scenario 7:*** Using the values shown in Table 1, the number of packets sent daily by each ED varies in a 365-day simulation time.
- ***Scenario 8:*** Unlike traditional ADR approaches, in this Scenario, we have studied both the impact of CR and CF on LoRa network performance as well as the impact of SF and TP.

Additionally, all simulation scenarios are evaluated in urban and sub-urban environments, which operate differently in terms of path loss, channel saturation, and simulation radius parameters as demonstrated in Table 2. The parameter values selected for the two environments being simulated are such that make our work directly comparable to other works on the relevant scientific field, e.g., [11,15,24,30].

## 6. Simulation Results

Through simulations, we compare our proposed algorithm with the ADR-MAX [4], ADR-AVG [11], No-ADR (“No-ADR” indicates the absence of ADR; ADR is disabled and is not being used in this scheme.), and ADR-Lite [15] schemes in the eight aforementioned scenarios.

### 6.1. Scenario 1: Performance under a Varying Number of Static EDs

**Figure 8:**
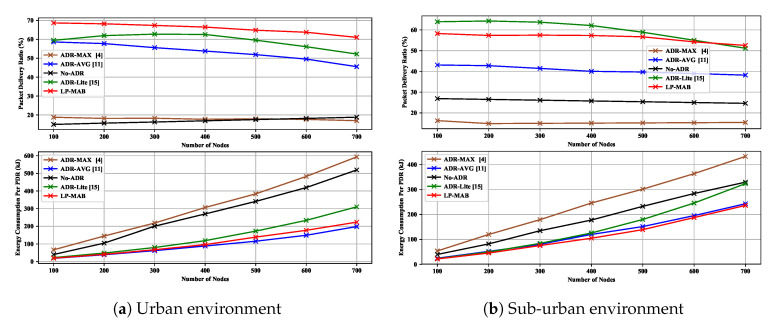
PDR & EC versus different numbers of static EDs in Scenario 1.

Figure 8a shows the PDR and EC in ADR-MAX, ADR-AVG, No-ADR, ADR-Lite, and LP-MAB in Scenario 1, for an urban environment with a radius of 480 m and σ equal to 3.56. As observed, the PDR of our proposed solution is higher than others due to the use of a combination of a short-term initial exploration phase and a long exploitation phase, which follows the exploration phase. Additionally to that, in many RL techniques, the initial action probability is defined as a uniform distribution, i.e., $P_{a}{}_{k}^{u}\left(t=0\right)=\frac{1}{\left|A\right|},\forall a{}_{k}^{u}\in A,u\in U$ [13]. Uniform probability initialization in such solutions as LoRa-MAB can take a long time to eliminate wrong choices from the actions, leading to increased convergence time. However, in LP-MAB, we do not assume an equal probability initialization for each action of an ED. Instead, we set the initial probabilities as an undefined number, i.e., nan, which can be changed based on the ACK reception as demonstrated in the initial state shown in Figure 4.

Keeping a long system history from the start until the present enables the proposed method to maintain a more comprehensive understanding of the network and achieve a better performance than other ADR mechanisms, which have only a history of the last twenty packets received. We also see in Figure 8a that, in low network densification (N<300), the LP-MAB’s EC is lower than other approaches due to the fact that instead of making a decision based on only a portion of the previously received packets, we made decisions based on the entire history of received packets. In this way, from the first received packet to the last one, the NS tries to find the most optimal action to improve the network’s performance.

In Figure 8b, we illustrate the PDR and EC in ADR-MAX, ADR-AVG, No-ADR, ADR-Lite, and LP-MAB in Scenario 1, for a sub-urban environment with a radius of 9800 m. It can be seen that, due to the greedy manner of decision-making in ADR-Lite, it is possible to achieve a higher level of PDR, regardless of the network density, at the cost of a higher EC. Also, in the No-ADR scheme, because of its randomness and its lack of consideration for environmental changes, the result is entirely dependent on the initial transmission parameter values. Therefore, the No-ADR scheme achieves a better performance in the sub-urban environment compared to the urban environment. As a result of applying machine learning techniques such as RL, the NS can converge to the optimal state in terms of TP, resulting in the lowest EC of LP-MAB compared to other approaches. Another pertinent observation from Figure 8 is that the reduction in PDR and EC performance associated with increasing the number of the EDs is negligible, making the LP-MAB approach more scalable than others.

### 6.2. Scenario 2: Performance under Varying Values of Channel Saturation

**Figure 9:**
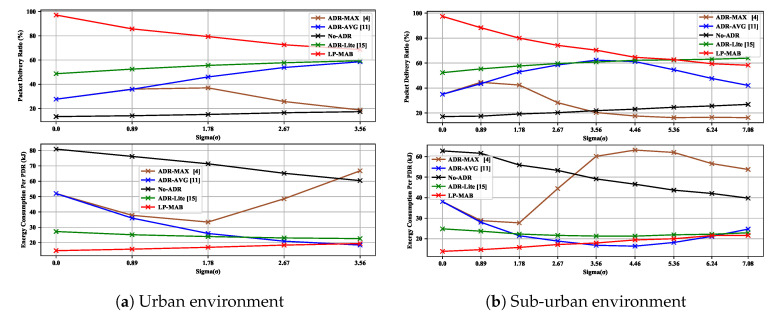
PDR & EC versus different values of channel saturation in Scenario 2.

In this Scenario, the channel noise, i.e., sigma (σ), takes the values of {0,0.89,1.78,2.67,3.56} and {0,0.89,1.78,2.67,3.56,4.46,5.36,6.24,7.08} for the urban and the sub-urban environment, respectively. In Figure 9, we illustrate the PDR and EC of different algorithms versus σ for the 100 static EDs used in Scenario 2. In the LP-MAB scheme, the reception of the NS’s ACKs by the EDs can directly influence the network’s performance. Thus, a higher rate of successful reception of the NS’s feedback by the EDs can contribute to a higher likelihood of determining the most optimal action.

Due to the lack of noise for σ=0, the EDs can receive most of the feedback, which can result in almost 100% PDR and the lowest EC for LP-MAB compared to other schemes, in both urban and sub-urban environments. Increasing channel noise decreases the probability of successful feedback reception, so sub-optimal actions are selected for the EDs, resulting in reduced PDR and an increase in EC. In contrast to LP-MAB, when σ increases, the ADR-MAX’s EC also increases significantly, especially in noisy channels, because, as σ increases, the ADR-MAX’s PDR decreases, thus causing the EDs to choose less optimal actions, resulting in an increase in the EC.

### 6.3. Scenario 3: Performance under a Varying Number of Mobile EDs

A wide variety of applications require or apply mobility enabled by the IoT. Mobile applications are found in traffic monitoring, smart metering, and animal tracking [31,32]. Through this Scenario, we are investigating the effects of mobility on the performance of EDs, by comparing various ADR mechanisms. In this work, we assess the use of the Random Waypoint Mobility Model for simulating LoRaWAN [32]. This Scenario runs for σ=7.08 and the number of EDs varies between 100 and 700 in both the urban and the sub-urban environment, while the EDs’ speed varies from zero to five meters per second and follows an exponential distribution.

**Figure 10:**
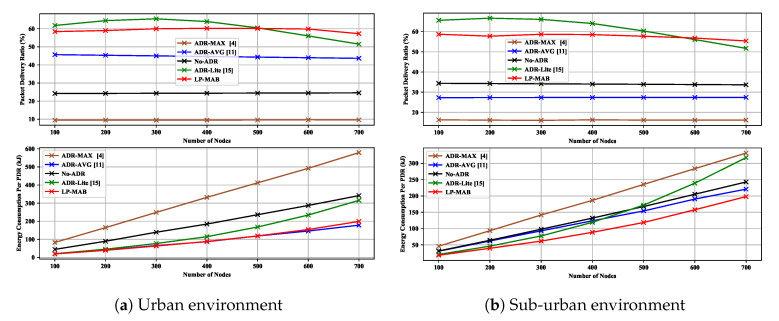
PDR & EC versus different numbers of mobile EDs in Scenario 3.

Figure 10 shows the PDR and EC in ADR-MAX, ADR-AVG, No-ADR, ADR-Lite, and LP-MAB in Scenario 3. In LP-MAB, unlike ADR-Lite, the PDR, in both the urban and the sub-urban environments, does not degrade as the network densification increases because of the LP-MAB’s scalability feature. Thus, our proposed algorithm outperforms other ADR mechanisms in terms of PDR as the number of EDs increases. Compared to all other methods, our proposed algorithm achieved the lowest EC in the urban environment for low ED densification, and in the sub-urban environment for any number of EDs. We can attribute this to the multi-reward technique we have in place in our scheme, which means that the actions with the highest TP receive the lowest reward.

### 6.4. Scenario 4: Performance under Varying Values of Speed for Mobile EDs

**Figure 11:**
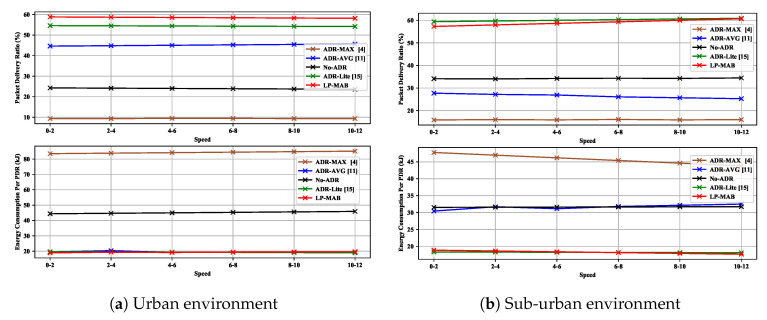
PDR & EC versus different values for speed for mobile EDs in Scenario 4.

For different IoT applications that require mobility, depending on the use case, the EDs may have varying speeds. For instance, in smart bicycles and animal monitoring applications, the EDs’ speed can be greater than 20 or lower than 5 km per hour, respectively. We examined the impact of different mobility speeds in a mobile Scenario, and the ways in which ADR mechanisms could be used to overcome the potential impact of mobile IoT devices. In this Scenario, EDs have a relatively low speed, between zero and twelve meters per second, with the speed varying in small steps of 2 m per second, i.e., of 7.2 km per hour, which is typical of IoT deployments in real-world environments. The varying speeds do not affect network performance in both the urban and the sub-urban environments, as can be seen in Figure 11.

### 6.5. Scenario 5: Performance under Varying Network Sizes and Different Mobility Speeds

**Figure 12:**
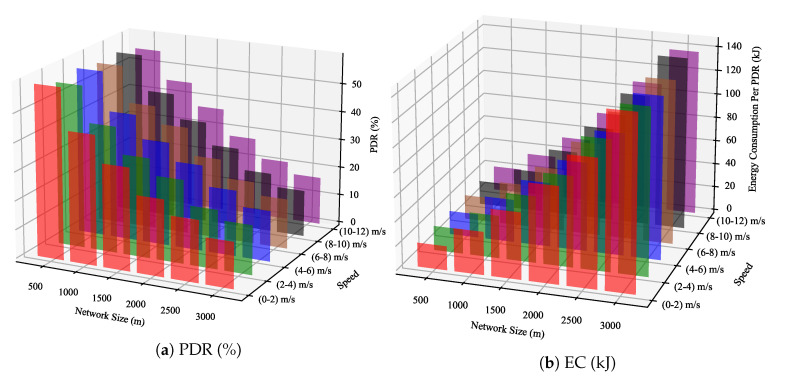
PDR & EC of the LP-MAB scheme versus varying network sizes and different mobility speeds in Scenario 5.

Figure 12 shows the PDR and EC of the LP-MAB scheme for a variety of network sizes (small and large network areas) using 100 EDs, for different mobility speeds, based on the Random Waypoint Mobility Model [32]. As can be seen in Figure 12, by increasing the network size, the overall performance of the network will be degraded, regardless of its speed, the same as indicated in Scenario 4. It is important to note that the configured path loss model in our work is LoRaLogNormalShadowin, which is appropriate for small area networks as [11], unlike the LoRaPathLossOulu path loss model used in [32], which is usually used in large area networks.

### 6.6. Scenario 6: Performance under a Varying Number of Simulation Days

**Figure 13:**
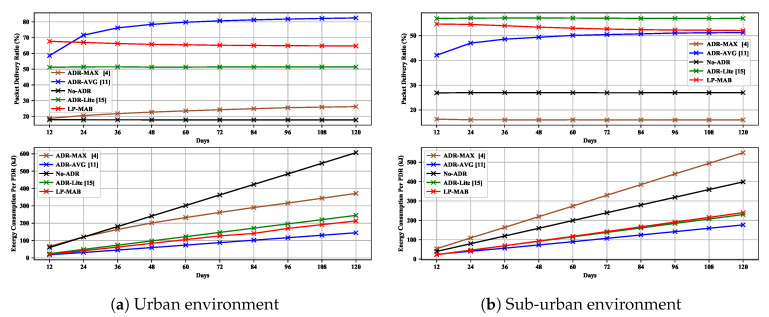
PDR & EC versus different numbers of simulation days in Scenario 6.

In Figure 13, we illustrate the PDR and EC of different algorithms versus the number of simulation days for the 100 static EDs used in Scenario 6. According to the results, ADR-AVG performs better than other ADR mechanisms in terms of PDR in urban environments, as well as in terms of EC in both urban and sub-urban environments. It should be noted that these performance results were achieved in a low-density deployment of EDs. This may be incongruous with most IoT applications, requiring several hundred EDs, for which, as demonstrated in Scenarios 1 and 3, the performance of ADR-AVG may not be satisfactory.

Additionally, LP-MAB’s results are consistently second-best in this Scenario in terms of both PDR and EC, and may outperform other algorithms, if more EDs are included in this simulation scenario. Thus, we note that there seems to exist a trade-off between the ED densification and the number of days being simulated.

### 6.7. Scenario 7: Performance under a Varying Number of Packets Sent per Day

**Figure 14:**
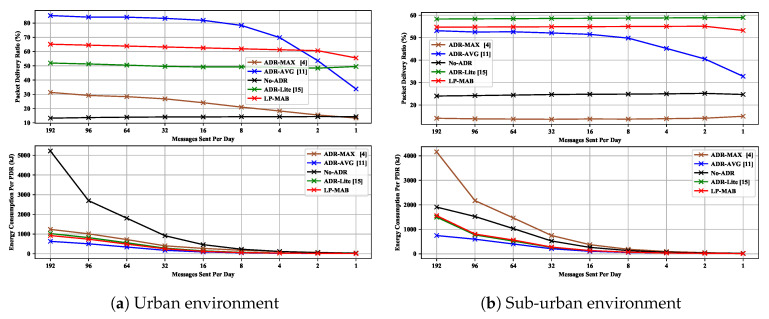
PDR & EC versus different values for number of sending message per day in Scenario 7.

Figure 14 shows the PDR and EC in ADR-MAX, ADR-AVG, No-ADR, ADR-Lite, and LP-MAB in Scenario 7 for 100 static EDs in a 365-day simulation time. As discussed in Scenario 6, in an urban environment with low densification, ADR-AVG can outperform other algorithms. By lowering the average number of daily sent packets per ED, we can observe a throughput degradation of those algorithms whose performance directly depends on the reception of the feedback from the NS, i.e., ADR-AVG and LP-MAB, which are making decisions based on the last 20 received packets and the history of all the last received packets, respectively. Among the examined schemes, ADR-Lite as a low-complexity scheme that decides the following action based only on the last received packet, and No-ADR as a basic randomly deciding algorithm that does not apply any specific decision-making approach, exhibit a performance that remains unchanged by the reduction of the daily sent packets in the network.

### 6.8. Scenario 8: Performance under a Varying Number of Total Actions Available

Similar to the novel Scenario examined in our other work [15], here, we are also evaluating the impact of increasing the state space of the transmission parameters over a 120-day simulation time. In this way, we provide more freedom of choice in configuring the transmission parameters while applying no changes to the protocol design and adding no overhead to the LoRa packet’s header. Based on the parameter values shown in Table 1, the EDs can choose the SF, TP, CF, and CR using the following values: {7,8,9,10,11,12}, {2,5,8,11,14}, {868.1,868.4,868.7}, and $\left\{\frac{4}{5},\frac{4}{6},\frac{4}{7},\frac{4}{8}\right\}$, respectively. In addition, it is important to note that as indicated in [33], BW cannot easily be altered due to the regularity limitations. Therefore, we have only considered the effects of SF, TP, CF, and CR with a cardinality of 6, 5, 3, and 4, respectively. For this Scenario, same as in [15], four different configurations were examined, namely Config-1, Config-2, Config-3, and Config-4, where the transmission parameters are: {SF+TP}, {SF+TP+CF}, {SF+TP+CR}, and {SF+TP+CF+CR}, respectively. Although, in real environments, the CF may not be adjustable for each ED, it can be modified during FLoRa simulations.

**Figure 15:**
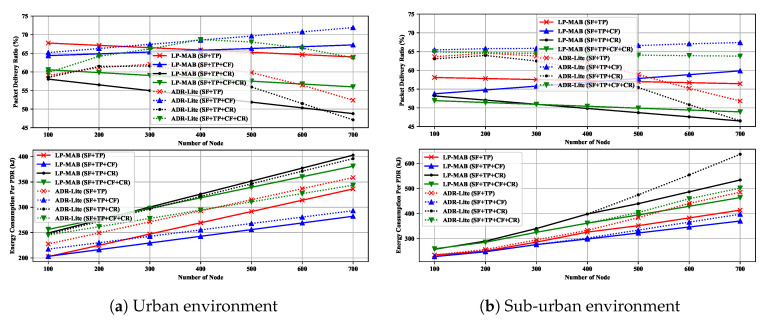
PDR & EC versus different values for number of total actions in Scenario 8.

Figure 15 shows that, contrary to the initial assumptions about the higher degree of freedom in the choice of transmission parameters, the possibility of increasing PDR in both urban and sub-urban environments is rather limited. Config-1 uses the SF and TP as transmission parameters, which is the default configuration parameter set for ADR mechanisms, resulting in the same result as in Scenario 1. In general, increasing the number of CFs can result in less collision probability in each frequency, since inter-SF collisions, which are an important factor affecting the network’s performance, will be less likely. Therefore Config-2, which allows EDs to select different CFs for packet transmission, performs better than any other configuration in terms of both PDR and EC.

According to Equation (8), by increasing the CR, the physical message length will also increase, which will result in a longer ToA duration and, consequently, an increase in the chance of collision occurrence and a higher EC. Thus, in Config-3, in which the parameters’ selection state space has increased by allowing for the use of higher CR values for packet transmission, the overall network performance will be reduced. Albeit using multiple channels, i.e., a higher SF, can improve network performance even when a higher CR, i.e., a more effective error correction code, is selected. Nevertheless, also in this case, the total overhead of the network will also grow higher, resulting in unsatisfactory performance, as seen in Config-4’s results.

As illustrated in Figure 15 the PDR degradation in Config-1, Config-3, and Config-4, as well as the PDR growth in Config-2, in both the urban and the sub-urban environments, is more consistent in the LP-MAB approach in comparison to the ADR-Lite scheme due to LP-MAB’s scalability feature as discussed in Scenario 1. In general, however, our results validate the results presented in [15] regarding the ADR-Lite scheme’s performance in the four different configurations examined, as well as the general performance of ADR schemes in the context of these four configurations.

## 7. Conclusions and Future Works

This article introduces a centralized adaptive configuration algorithm to improve the PDR and EC in the context of LoRaWan, as these two metrics constitute the main performance metrics for LoRa networks. For this goal, we have presented an RL-based ADR algorithm that allows the NS to configure the EDs’ transmission parameters. This algorithm, which we have named LP-MAB, can achieve an efficient adaptive configuration using two MAB algorithms, SE and EXP3, after mapping the LoRa resource allocation problem to the MAB problem. By using SE and EXP3, the proposed solution can simultaneously benefit from the advantages of a short-term initial exploration phase and of a long exploitation phase, which follows the exploration phase.

Using several scenarios, we have evaluated the performance of the LP-MAB and compared it with other ADR mechanisms, namely the ADR-MAX [4], ADR-AVG [11], No-ADR, and ADR-Lite [15], in different circumstances. The simulation results indicate that the LP-MAB’s EC outperforms other algorithms while maintaining a relatively high PDR in various circumstances. LP-MAB is also more scalable than other approaches, since its PDR decreases relatively slowly as the number of the EDs increases. In IoT applications, where the battery lifetime is the most important factor, LP-MAB makes LoRa one of the best candidates for adoption as the main communication protocol among the vast number of EDs deployed.

As LoRa utilizes the ALOHA protocol as its Media Access Control (MAC) mechanism, the consequent dynamic value for the number of potential re-transmissions may lead to an increased PDR. Therefore, as a future work, we propose the use of an RL-based algorithm to specify the number of potential re-transmissions, which should be able to take into account the overall environmental conditions of the LoRa network, including the number of the EDs, the relevant noise, and the network size, on the one hand, as well as the trade-off between the number of potential packet re-transmissions and the EC, on the other hand.

## Figures and Tables

**Table 1:** Simulation Setup Parameters.

Parameter	Value
Simulator Platform	OMNet++
Simulator Model	INET and FLORA
Repetitions	20
Mobility Model	Random Waypoint
ED Speed (*v*)	{0,…,12} m/s
Urban Cell Radius (*r*)	480 m
Sub-urban Cell Radius (*r*)	9800 m
Packet Length (*L*)	20 bytes
BW (BW)	125 kHz
Simulations Time (*T*)	12 days
Number of EDs (*N*)	{100,…,700}
Number of GWs (#GW)	{1,…,10}
Urban Environment’s Sigma (σ)	{0.0,…,3.56}
Sub-urban Environment’s Sigma (σ)	{0.0,…,7.08}
SFs (SF)	{7,8,9,10,11,12}
TPs (TP)	{2,5,8,11,14} dBm
CFs (CF)	{868.1,868.4,868.7} MHz
CRs (CR)	$\left\{\frac{4}{5},\frac{4}{6},\frac{4}{7},\frac{4}{8}\right\}$
Number of Sent Packets per Day (ϵ)	{1,…,192} packets/day

**Table 2:** Standard deviation of the path loss (σ) in dB and other parameters for the different deployment scenarios. This table is partially adapted from [30] and based on the relevant values provided in [23,30].

Scenarios	$d_{0}$ [m]	$\bar{PL}\left(d_{0}\right)$ [dB]	*n*	σ [dB]	Cell Radius (*r*) [m]
Urban	40	127.41	2.08	3.57	480
Sub-urban	1000	128.95	2.32	7.08	9800

## Data Availability

The FLoRa-based framework for simulating LP-MAB is available at https://github.com/reza-serati/LP-MAB (accessed on 11 January 2023).

## Abbreviations

The following abbreviations are used in this manuscript:

3G	third-generation cellular network
4G	fourth-generation cellular network
ACK	ACKnowledgment
ADR	Adaptive Data Rate
BW	BandWidth
CF	Carrier Frequency
CR	Coding Rate
CRC	Cyclic Redundancy Check
CSMA/CA	Carrier-Sense Multiple Access with Collision Avoidance
CSS	Chirp Spread Spectrum
EC	Energy Consumption
ED	End Device
EXP3	EXPonential weights for EXPloration and EXPloitation
FLoRa	Framework for LoRa simulations
GW	GateWay
IoT	Internet of Things
km	kilometers
LoRa	Long Range
LoRaWAN	Long-Range Wide-Area Network
LP-MAB	Low-Power Multi-Armed Bandit
LPWAN	Low-Power Wide-Area Network
m	meters
MAB	Multi-Armed Bandit
MAC	Media Access Control
ML	Machine Learning
NB-IoT	NarrowBand IoT
NS	Network Server
OWA	Ordered Weighted Averaging
PDF	Probability Density Function
PDR	Packet Delivery Ratio
RL	Reinforcement Learning
SE	Successive Elimination
SINR	Signal-to-Interference-plus-Noise Ratio
SF	Spreading Factor
ToA	Time-on-Air
TP	Transmission Power
